# The Prevalence of Osteoporosis and Osteopenia Among Older Adults in a Community-Based Setting in Riyadh, Saudi Arabia

**DOI:** 10.7759/cureus.32765

**Published:** 2022-12-21

**Authors:** Muath Alkhunizan, Nouf Almasoud, Majd Munia Abdulmowla, Zoha Khalid

**Affiliations:** 1 Family Medicine, King Faisal Specialist Hospital & Research Centre, Riyadh, SAU; 2 Medicine, Alfaisal University, Riyadh, SAU; 3 Clinical Research, King Faisal Specialist Hospital & Research Centre, Riyadh, SAU

**Keywords:** older adults, geriatric, saudi arabia, prevalence, osteopenia, osteoporosis

## Abstract

Background

Osteoporosis is a disease of bone density. It makes bones fragile and easy to break. Fragility fractures can cause significant morbidity, mortality, poor quality of life, and financial stress on medical care. Osteoporosis is overlooked and undertreated. Saudi Arabia issued a national plan promoting the early diagnosis and management of osteoporosis. Few and old studies are available in Saudi Arabia estimating the prevalence of osteoporosis in the Saudi older adult population. The aim of this study is to measure the prevalence of osteoporosis and osteopenia among older adult patients.

Methodology

This retrospective cross-sectional study is based on data gathered from patients ≥60 years of age. Data were collected from January 1, 2016, to December 31, 2021, for patients who were attending family medicine clinics at King Faisal Specialist Hospital & Research Centre in Riyadh, Saudi Arabia, who were screened using dual-energy X-ray absorptiometry (DEXA). Patients who have secondary causes of osteoporosis were excluded.

Results

A total of 1,302 patients were studied during the course of data collection. The mean age was 68.26. Out of the studied subjects, 75% were female and 25% were males. The prevalence of osteoporosis was 8.2% and 11.8% in femoral and lumbar bone mineral density (BMD) results, respectively. The prevalence of osteopenia based on femoral and lumbar BMD results was 50.2% and 41.2%, respectively.

Conclusion

Osteoporosis and osteopenia are prevalent in the Saudi older adult population. Multiple clinical characteristics have been associated with low bone density disease. Thus, it is important to reinforce primary care physicians’ efforts for early screening and treatment of the Saudi older adult population based on their clinical and demographic risk factors.

## Introduction

Low bone mineral density (BMD) can be manifested as osteoporosis or osteopenia [1]. Osteoporosis and osteopenia are skeletal disorders that make bones fragile and more prone to fractures, ultimately leading to major disabilities and poor quality of life [2]. In a recently published meta-analysis, the prevalence of osteoporosis worldwide was found to be 18.3%, with the highest prevalence reported in Africa [3]. In the United States (USA) and Europe, osteoporosis affects about 30% of all postmenopausal women [3].

In Saudi Arabia, a few studies have estimated the prevalence of osteoporosis among adults ranging from 23.4% to 39.5%; however, most of these studies date back more than 15 years [4-7]. The most recent estimate of osteoporosis in Saudi Arabia is 52.8% of women and 63.6% of men in a hospital-based setting [8]. Around 200 million people over the world have osteoporosis, causing more than 8.9 million fractures yearly [9]. The incidence of femur fractures caused by osteoporosis is approximately 4,950/100,000 people-year for all individuals in Saudi Arabia [10].

Osteoporosis is linked to a range of terrible health outcomes in patients, including their physical, mental, and emotional well-being [11]. Fragility fractures are associated with higher rates of morbidity and mortality [12]. Poor quality of life, following a fragility fracture, stems from possible symptoms including chronic pain from fractures, limitation of basic activity of daily living, or reduced mobility [12,13].

From a financial standpoint, a recent study in Saudi Arabia estimated the mean direct medical cost for osteoporosis of patients without fractures as 975.77 USD per person per year (PPPY) and for patients with fractures as 9,716.26 USD PPPY, which might be an underestimate as the data reflects governmental hospital pricing [14]. One study developed a model that estimated osteoporotic fractures amounting to 988,029 within a five-year period from 2019 to 2023. The cost of burden was extrapolated to be 13.5 billion SR (3.59 billion USD) within this five-year period [15]. As the prevalence of osteoporosis and fragility fractures rise due to the aging Saudi population, the economic burden incurred by osteoporosis is expected to increase in the coming years [16].

Given the huge impact on medical and financial conditions, it is frequently overlooked and undertreated by primary healthcare providers [17]. Early detection and prompt treatment are critical to avoid further complications [18]. The United States Preventive Services Task Force recommends screening for osteoporosis for women 65 years and older and postmenopausal women younger than 65 years with risk factors [19]. The Saudi national plan issued by the Ministry of Health in 2018 encouraged primary care physicians to screen patients at high risk of osteoporosis, anyone above the age of 50 years, postmenopausal women, and those with a history of fragility fracture [13]. The discrepancy in guidelines of screening stems from the high prevalence of osteoporosis and fragility fractures at an earlier age in the Saudi population. The prevalence of osteoporosis in women and men above the age of 50 was recorded as 28.2% and 37.8%, respectively, in a study done on 1,980 randomly selected adults in Saudi Arabia [5].

## Materials and methods

Study design and setting

This retrospective cross-sectional study was conducted on patients attending family medicine and polyclinics at King Faisal Specialist Hospital & Research Centre in Riyadh, Saudi Arabia. Data were collected between January 1, 2016, and December 31, 2021.

Ethical approval

This study was approved by the Institutional Review Board committee at King Faisal Specialist Hospital & Research Centre. The information of the subjects enrolled in this research remained confidential, and their privacy was assured.

Inclusion criteria and exclusion criteria

Data were gathered from patients aged 60 years and older who were screened for osteoporosis using dual-energy X-ray absorptiometry (DEXA). Patients who have secondary causes of osteoporosis such as liver, kidney, and malabsorption diseases were excluded.

Data collection and variables

The electronic records of 1,302 subjects were collected. Data were collected in Microsoft Excel (Microsoft Corp., Redmond, WA, USA) by two medical graduates based on the data documented by the treating physician of the patient. The variables recorded included age in years, sex, educational level, marital status, body mass index (BMI), current smoking status, corticosteroid usage, previous fracture, family history of fracture, and past medical history of rheumatoid arthritis, diabetes, hypertension, and vitamin D deficiency.

BMD measurement and interpretation

All enrolled subjects were evaluated using DXA (Hologic Inc., Marlborough MA, USA) at the lumbar spine and femoral neck bone mass density (BMD). Results were expressed as T-scores and compared to ones obtained in a gender-matched Caucasian population at the peak of bone mass [20]. Based on the World Health Organization (WHO) reference, a T-score of less than or equal to 2.5 standard deviation (SD) at any skeletal site was defined as osteoporosis, whereas osteopenia was defined as a T-score between -1 and -2.5 SD. Scores of -1 and above were considered normal.

BMI calculation and interpretation

BMI was calculated by dividing weight in kilograms with height in meters squared (BMI = kg/m^2^). This calculation has been recorded in the electronic record automatically after the height and weight of the patient are registered before the visit. BMI under 18.5 kg/m^2^ was considered underweight. BMI between 18.5 kg/m^2^ and under 24.9 kg/m^2^ was considered normal. BMI above 25 kg/m^2^ and under 29.9 kg/m^2^ was considered overweight. A BMI of 30 kg/m^2^ and above is considered obese.

Statistical analysis

The Statistical Package for the Social Sciences (SPSS) version 24.0 (IBM SPSS Statistics, Armonk, NY, USA) was used for all statistical analyses. A p-value ≤ 0.05 was considered statistically significant. Descriptive statistics are presented as a p-value < 0.05 with a 95% confidence interval (CI), continuous data as mean ± SD, and categorical data as frequency and percentage. Chi-squared and Fisher’s exact tests for categorical variables and independent t-test for continuous variables were used.

## Results

Baseline characteristics of the study subjects

We studied the data of 1,302 subjects, whose mean age was 68.26 (SD: 6.902) years. Among them, 977 subjects were female, accounting for 75% of subjects, and 325 were male, accounting for 25%. Almost 55% of the studied subjects were obese based on their BMI, 30.8% were overweight, and 13.7% were normal weight. In terms of medical comorbidities, 56.5% were hypertensive, 51.7% were diabetic, 20.4% had hypothyroidism, 5.1% had vitamin D deficiency, 5.5% were using corticosteroids, and 4.1% had previous fractures (Table 1).

**Table 1:** Demographics SD: standard deviation

Variable		Mean (± SD)	
Age		68.26 (± 6.902)	
		Number	%
Gender	Male	325	25%
	Female	977	75%
BMI	Underweight	9	0.7%
	Normal	176	13.7%
	Overweight	396	30.8%
	Obese	705	54.8%
Marital status	Single	34	2.6%
	Married	1,112	85%
	Widow	151	11.6%
Current smoking	Yes	16	1.2%
	No	1,286	98.8%
Diabetes	Yes	673	51.7%
	No	629	48.3%
Rheumatoid arthritis	Yes	11	0.8%
	No	1,291	99.1%
Hypertension	Yes	735	56.5%
	No	567	43.5%
Vitamin D deficiency	Yes	66	5.1%
	No	1,236	94.9%
Corticosteroid use	Yes	71	95.5%
	No	1,231	94.5%
Hypothyroidism	Yes	266	20.4%
	No	1,036	79.6%
Renal disease	Yes	40	3.1%
	No	1,263	96.9%
Liver disease	Yes	22	1.7%
	No	1,281	98.3%
Neoplasm	Yes	22	1.7%
	No	1,281	98.3%
Previous fracture	Yes	53	4.1%
	No	1,249	95.9%
Family history of hip fracture	Yes	2	0.2%
	No	1,300	99.8%

Low BMD prevalence in relation to sociodemographic characteristics

The prevalence of osteoporosis was 8.2% and 11.8% in femoral and lumbar BMD results, respectively. The prevalence of osteopenia based on femoral and lumbar results was 50.2% and 41.2%, respectively (Table 2).

**Table 2:** BMD results BMD: bone mineral density

Variable		Number	%
Diagnosis based on femoral BMD	Normal	540	41.5%
	Osteopenia	654	50.2%
	Osteoporosis	107	8.2%
Diagnosis based on lumbar BMD	Normal	612	47%
	Osteopenia	536	41.2%
	Osteoporosis	154	11.8%

**Figure 1:**
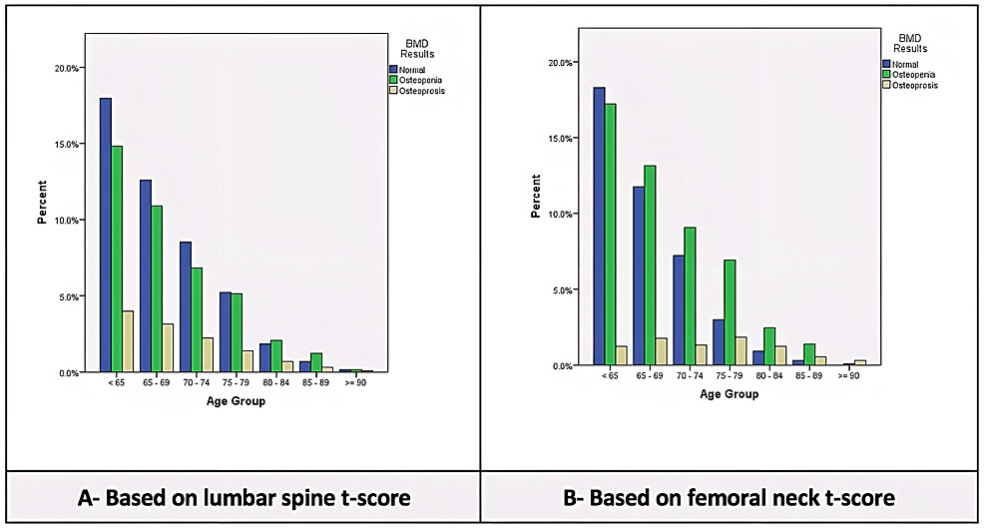
Age-specific prevalence BMD: bone mineral density

Osteoporosis was reported more in patients who are 75-79 years of age with a percentage of 3% based on the lumbar spine, whereas a higher percentage was reported in patients 59-65 years of age with a percentage of 4% based on the femoral neck. Osteopenia was reported more in patients who are 59-65 years of age in both the femoral and lumbar spine (Figure 1A and Figure 1B).

**Figure 2:**
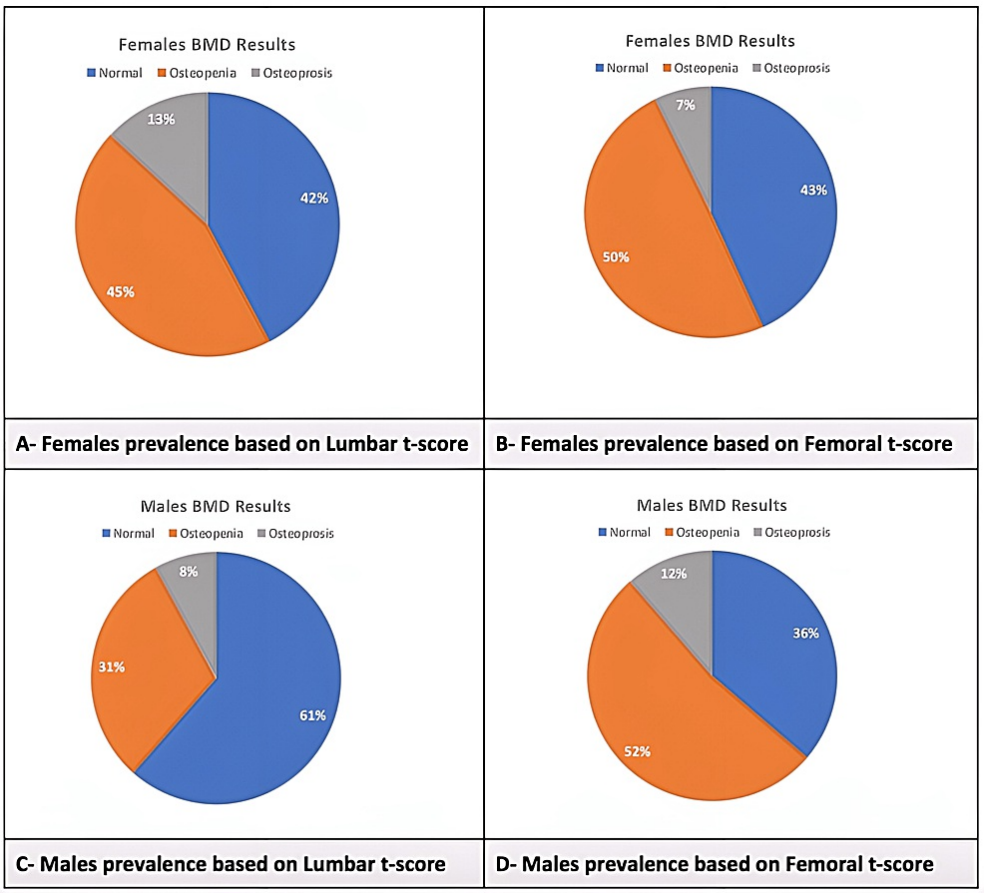
Prevalence according to sex BMD: bone mineral density

Regarding the prevalence of osteoporosis and osteopenia based on the sex of patients, in females, it was 13% and 7% for femoral and lumbar BMD results, respectively. Of the females, 45% and 50% have osteopenia based on femoral and lumbar BMD results, respectively. For males, 8% and 12% have osteoporosis based on femoral and lumbar BMD results, respectively. Osteopenia was found to be 45% and 50% in females, whereas in males, it was 31% and 52% based on femoral and lumbar BMD results, respectively (Figure 2A-2D).

Association of clinical and sociodemographic characteristics with low BMD

Age, sex, BMI, diabetes, hypertension, and corticosteroid use were strongly associated with osteoporosis in our study (p-value < 0.05). Other variables showed no statistically significant association (Table 3).

**Table 3:** Distribution of patients with osteoporosis with reference to other variables SD: standard deviation, BMI: body mass index

Variable		Diagnosis of osteoporosis (mean (± SD) or %)		p-value
		Yes	No	
Age		68.68 (± 7.309)	68.20 (± 6.847)	<0.05
BMI		28.72 (± 6.06)	31.33 (± 6.351)	<0.0001
Sex	Female	83.1%	74%	<0.05
	Male	16.9%	26%	
Diabetes	Yes	37.7%	53.6%	<0.001
	No	62.3%	46.4%	
Hypertension	Yes	40.9%	58.5%	<0.0001
	No	59.1%	41.5%	
Vitamin D deficiency	Yes	6.5%	4.9%	>0.05
	No	93.5%	95.1%	
Corticosteroid use	Yes	1.9%	5.9%	<0.05
	No	98.1%	94.1%	
Hypothyroidism	Yes	10.4%	21.8%	>0.001
	No	89.6%	78.2%	
Renal disease	Yes	1.9%	3.2%	>0.05
	No	98.1%	96.8%	
Liver disease	Yes	1.3%	1.7%	>0.05
	No	98.7%	98.3%	
Neoplasm	Yes	0.6%	1.8%	>0.05

## Discussion

The purpose of our study is to gain more insight into and understanding of the prevalence of osteoporosis and osteopenia among the geriatric population; the prevalence of osteoporosis was 8.2% and 11.8% based on femoral and lumbar results, respectively. When comparing our data to previously published articles in Saudi Arabia, a recently published article by Sadat-Ali et al. showed the prevalence to be 52.8% in women and 63.6% in men using the T-score of the lumbar spine [8]. AlQuaiz et al. reported that screening of 362 healthy women using DEXA revealed that 58.6% had low bone density; however, this group comprised only women between 40 and 50 years old [21]. Gouhar et al. reported osteoporosis prevalence to be 29.7% and 22% in people aged 60 years and above [22]. Ardawi et al. found osteoporosis at the lumbar spine to be 38.3%-47.7% for Saudi patients aged 50-79 years who are living on the west coast [5]. The study by Ali Farsi et al. showed the prevalence of osteoporosis to be 9.3%; however, only 40% of their healthy population were in the 50-54 years age range, and their study was conducted on male subjects only [23].

Our study found that osteopenia prevalence was 50.2% and 41.2% based on femoral and lumbar BMD results, respectively. When comparing our data to previously published articles in Saudi Arabia, El-Desouki et al. reported that 30.6% of their subjects were found to have osteopenia, 27% with osteopenia in patients who are 60-69 years, and 21.5% of patients who are 70-79 years; however, their data were only on females [6]. Ali Farsi et al. reported osteopenia prevalence in Saudi males above the age of 50 years to be 40.7% [23]. Sadat-Ali et al. found osteopenia in postmenopausal females with an average age of 57.6 years to be 31.6% [24].

There is a discrepancy in prevalence results, especially in osteoporosis between our study and the previous studies, which could be explained by variations in sample size, subject characteristics, study setting, and BMD purpose when performed for subjects. Some previous studies have included only postmenopausal women or enrolled subjects from secondary or tertiary care settings.

Numerous risk factors have been previously identified to escalate osteoporosis or osteopenia development in an individual, for example, age, with its metabolism and the chronic loss of bony architecture with the aging process; female gender, as estrogen holds a protective effect on bone metabolism, especially the hormonal disturbance in postmenopausal status; medication use, such as corticosteroid use, which has been linked to loss of bone architecture; chronic diseases such as diabetes, rheumatoid arthritis, and hyperparathyroidism with different suggested pathophysiology; and vitamin D deficiency, as vitamin D is important for calcium absorption, which is essential for bone mineralization [1].

In our study, we established a significant association between osteoporosis and some variables, such as age, sex, BMI, diabetes, hypertension, and corticosteroid use. Other variables such as vitamin D deficiency, hypothyroidism, renal disease, liver disease, and neoplasm were not associated with osteoporosis in our results. Age, gender, and BMI are well-established risk factors in literature, supported by many studies [19,21-23], which is analogous to our results. On the other hand, vitamin D deficiency in our study showed no significant association with osteoporosis, which contradicts previous literature [19,21-23]. This could be contributed to the low rate of vitamin D deficiency reported in our patients, explained by the high rate of vitamin D level testing in our population and subsequently correctable results in our data.

Our study has limitations, especially for such retrospective studies as missing information in charts and certain variables may have not been reported. Furthermore, not all confounders were accounted for in our study. Our study’s strengths are the large number of subjects and that it is in a community-based setting.

## Conclusions

Osteoporosis and osteopenia are prevalent in the geriatric population in Saudi Arabia. Our study is the first community-based study in Saudi Arabia to shed a light on the prevalence of osteoporosis and osteopenia in the Saudi older adult population. Further studies will facilitate policymakers as well as healthcare managers to make decisions. Emphasis on primary care physicians to follow local and international guidelines when it comes to screening, diagnosing, and eventually treating osteoporosis is needed. Furthermore, we call for local campaigns for knowledge dissemination and awareness in the country of Saudi Arabia.
